# The cGAS-STING pathway in cancer: friend or foe

**DOI:** 10.1038/s41419-026-08607-2

**Published:** 2026-03-25

**Authors:** Qian Li, Qingkang Song, Lingli Ma, Kuan Kang, Xiaoru Zhu, Yuan Li, Xin Lin, Xingxing Lu, Zhaoyang Zeng, Guiyuan Li, Wei Xiong, Bo Xiang, Pan Chen, Mei Yi

**Affiliations:** 1 NHC Key Laboratory of Carcinogenesis and Hunan Key Laboratory of Cancer Metabolism, Hunan Cancer Hospital and the Affiliated Cancer Hospital of Xiangya School of Medicine, Central South University, Changsha, China; 2 FuRong Laboratory, Changsha, China; 3 Cancer Research Institute and School of Basic Medical Sciences, Central South University, Changsha, China; 4 The Key Laboratory of Carcinogenesis and Cancer Invasion of the Chinese Ministry of Education, Central South University, Changsha, China; 5 Department of Dermotology, National Clinical Research Center for Geriatric Disorders, Xiangya Hospital, Central South University, Changsha, China

**Keywords:** Cancer, Tumour immunology

## Abstract

The cGAS-STING pathway is crucial for recognizing aberrant DNA in the cytoplasm and activating the innate immune response. After detecting aberrant DNA in the cytoplasm, cGAS can catalyze the synthesis of cGAMP from ATP and GTP, which acts as a second messenger to engage STING and unleash type I interferons, thereby eliciting a robust antitumor immune cascade. In recent years, the role of the cGAS-STING pathway in tumor immunity has attracted widespread attention. Paradoxically, it not only activates antitumor immune responses but also promotes tumor progression under certain circumstances. This review untangles the safeguards that prevent cGAS from recognizing nuclear self-DNA, delineates the antitumor and pro-tumor mechanisms of the cGAS-STING axis, and surveys tumor immunotherapy strategies targeting this pathway.

## Facts

1. The contextual duality of the cGAS-STING pathway remains a central paradox. Determining the precise molecular and cellular conditions (e.g., activation strength, duration, cellular origin) that dictate whether it elicits antitumor immunity or promotes protumorigenic inflammation—and systematically dissecting the relative contribution of downstream branches (type I IFNs, non-canonical NF-κB, senescence programs) within specific tumor contexts—is a major prerequisite for its successful therapeutic exploitation.
2. Within the intricate tumor microenvironment, the functional consequences of immune cells response to tumor-intrinsic cGAS-STING activation and of cGAS-STING signaling within immune cells themselves remain poorly delineated. Future studies are needed to dissect the compartment-specific functions of this pathway and the consequences of its intercellular cross-talk.
3. Clinical trials of STING-agonist monotherapy have yielded limited efficacy; maximizing translational benefit will require next-generation agonists engineered to overcome poor delivery and rapid clearance, coupled with rational combination regimens that overcome therapeutic resistance.
4. Mechanisms that confine nuclear cGAS to prevent autoimmunity are emerging, yet whether its non-canonical functions—chromatin remodeling and transcriptional regulation—suppress or promote tumorigenesis independently of STING signaling remains an open and compelling frontier.

## Introduction

With long-term coexistence with various pathogens, such as viruses, bacteria, and parasites, the host has evolved two sets of defense and clearance mechanisms against pathogenic microorganisms. In addition to the precise targeting of adaptive immunity, innate immunity, also known as nonspecific immunity, serves as the first line of defense and plays a crucial role in the recognition and elimination of pathogens. In innate immunity, immune cells recognize specific components of pathogens, such as nucleic acids or cell membranes, known as pathogen-associated molecular patterns (PAMPs), through pattern recognition receptors (PRRs). A series of intracellular signaling pathways is subsequently activated, including the pro-inflammatory cytokine pathway, which is dependent on nuclear factor kappa B (NF-κB), and the type I interferon(IFN-Ⅰ) pathway, which is dependent on interferon regulatory factors (IRFs) [1]. This ultimately leads to the release of pro-inflammatory cytokines or IFN-I, which recruit more immune cells to participate in the clearance of viruses or bacteria. In antiviral immunity, the recognition of viral nucleic acids is particularly important. The first discovered intracellular RNA sensor was the RIG-I-like receptors (RLRs), which act as PRRs that recognize RNA, activating the expression of IFN-I [2]. In the search for DNA sensors, STING, located in the endoplasmic reticulum, is located upstream of the NF-κB and IRF pathways [3]. It can receive signals from the second messenger cGAMP produced by the cytoplasmic DNA sensor cGAS, thereby triggering subsequent immune responses [4]. Numerous studies have shown that this pathway not only plays an important role in antiviral infections but also participates in the occurrence and development of various diseases. In-depth research on this pathway is not only conducive to obtaining a deeper understanding of the host’s innate immune mechanisms but also beneficial for the precise treatment of diseases.

**Fig. 1:**
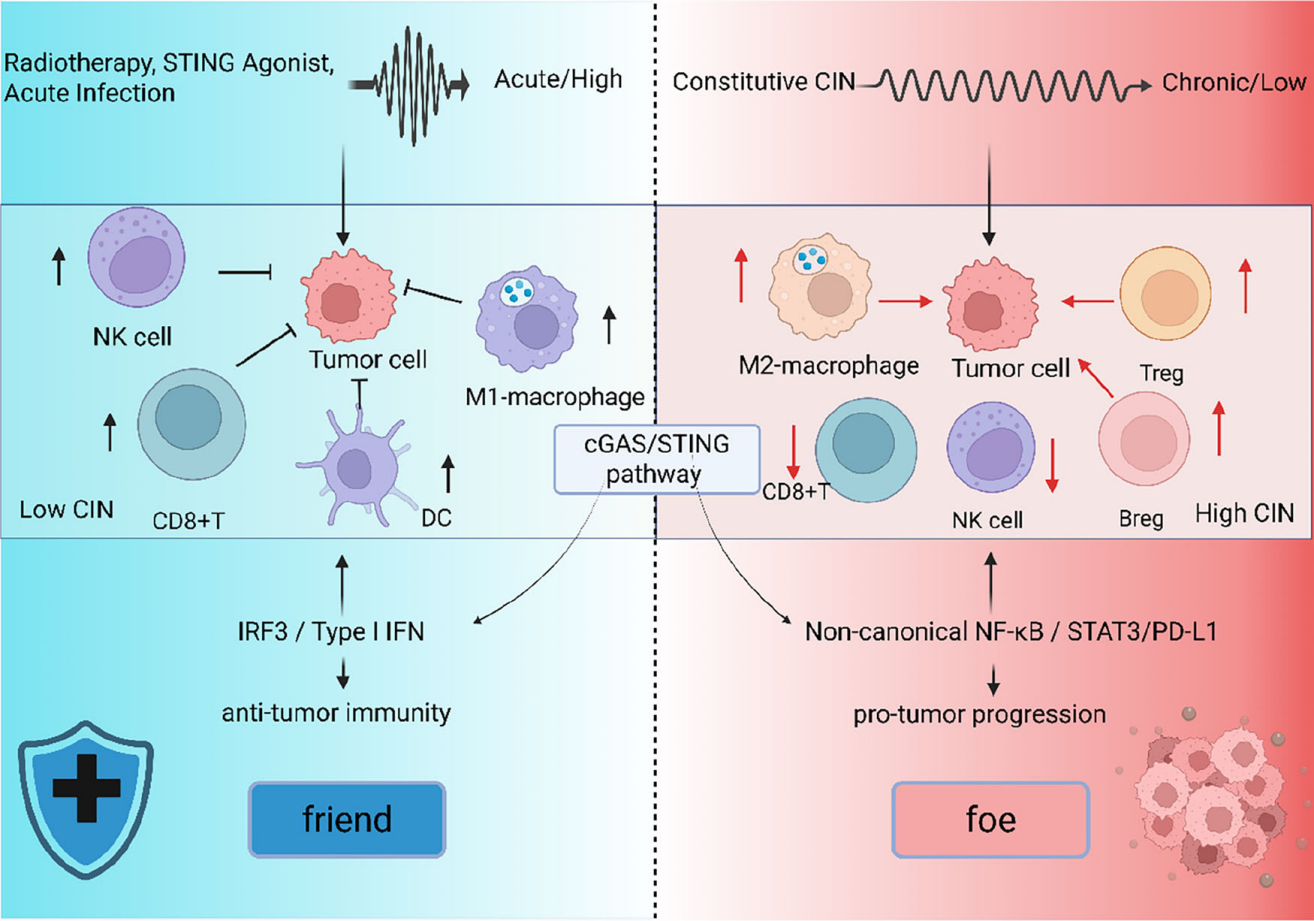
A context-dependent regulatory model of the cGAS/STING pathway in tumor immunity. The activation of the cAS-STING pathway in the tumor microenvironment can lead to divergent immunological outcomes depending on the context of activation. Key factors that determine the outcome include the strength, duration, and cellular origin of cGAS-STING activation, as well as the tumor microenvironmental context. Left: Under conditions of acute, high-level activation—such as induced by radiotherapy or exogenous STING agonists—the pathway preferentially induces IRF3 and type I interferon signaling, promoting dendritic cell (DC) maturation, CD8⁺ T cell activation, and M1-like macrophage polarization, thereby fostering antitumor immunity. Right: Conversely, chronic or low-level activation—often driven by constitutive chromosomal instability (CIN) in tumor cells—leads to sustained signaling through non-canonical NF-κB and STAT3, resulting in increased PD-L1 expression, recruitment of regulatory T cells (Treg), Breg, and M2-like macrophages, which collectively suppress immune responses and promote tumor progression. Figure created with BioRender.

In the field of oncology, a central and unresolved scientific question is becoming ever more pressing: within the complex and dynamic disease microenvironment, which key factors determine whether the cGAS-STING pathway ultimately exerts a protective, beneficial effect or a detrimental, pathological one? The decision logic governing this “friend-or-foe” switch is critical. To provide a unifying analytical lens for the entire review, we propose in Fig. 1 an integrative, context-dependent decision framework. The framework posits that the final functional output of the cGAS-STING pathway is not dictated by any single parameter; instead, it is the combinatorial and precise regulation of multiple dimensions—signal strength and spatiotemporal pattern, the specific cell type, local microenvironment, and the selective engagement of downstream branch pathways.

This article will review the activation process of this pathway and present the strategies by which cGAS prevents self-activation. Simultaneously, centered around the aforementioned framework, it will systematically dissect the dual role mechanisms of this pathway in cancer and its targeted therapeutic strategies.

## Activation of the cGAS‒STING pathway

After the discovery that DNA transfection or DNA viral infection in mammalian cells can trigger the production of cGAMP as a second messenger to activate subsequent immune pathways [5], Sun [4] identified the enzyme responsible for cGAMP synthesis—cGAS—through biochemical fractionation and quantitative mass spectrometry, which demonstrated that, in addition to bacteria, archaea, and protozoa, vertebrates also possess an evolutionarily conserved enzyme capable of synthesizing cyclic dinucleotides. Initially, cGAS was found to be predominantly located in the cytoplasm (rarely in the nucleus or perinuclear region), where it acts as a DNA sensor and synthesizes the second messenger cGAMP from ATP and GTP. cGAMP binds to the protein STING, activates interferon regulatory factor 3 (IRF3), promotes its dimerization and nuclear translocation, and thereby induces the production of IFN-I [4]. Subsequent studies revealed that cGAS is present not only in the cytoplasm but also in other subcellular structures, such as the cell membrane [6] and even the nucleus [7, 8]. However, the mechanism by which cGAS in the nucleus is not activated is related to the structure of nucleosomes in chromosomes [9, 10]. The activation of cGAS is strongly dependent on the length of the DNA [11], with the shortest DNA sequence required to activate human cGAS being 45 bp. Moreover, compared with mouse cGAS, human cGAS has two amino acid substitutions in the DNA-binding domain, resulting in a preference for longer DNA [12]. The activity of cGAS is regulated in various ways, including enhancing the binding of cGAS to DNA through cofactors to increase its activity or affecting its enzymatic activity through various post-translational modifications [13]. In addition, intrinsic substitutions in the human cGAS protein sequence can also regulate the activity of this pathway [12]. The direct sensing of double-stranded DNA by cGAS can produce a large amount of cGAMP, amplifying the signal level and enabling cells to detect and respond sensitively to minimal amounts of DNA in the cytoplasm, thereby triggering an immune response and maintaining the health of the organism. cGAS is capable of sensing not only intracellular DNA from foreign pathogens but also self-damaged DNA, unstable genomes in tumor cells, and endogenous mitochondrial DNA. Therefore, further research has revealed that this pathway also plays an important role in processes such as cellular senescence, death, and carcinogenesis. In addition, certain retroviruses [14] and parasites [15] can also activate this immune pathway.

The protein STING, located downstream of cGAS, can specifically recognize the signal of cyclic di-GMP derived from bacteria, thereby activating the IFN-I pathway [3, 16]. Cyclic di-GMP, which was originally thought to exist only in bacteria, is recognized by host cells as an ideal PAMP. Inspired by this, it has been discovered that mammalian cells can spontaneously produce a cyclic dinucleotide—2,3’-cGAMP—when infected with pathogenic DNA [17]. Unlike bacterial cyclic di-GMP, which is connected by two 3’-5’ phosphodiester bonds, cGAMP is formed by ATP and GTP connected through a 2’-5’ phosphodiester bond and a 3’-5’ phosphodiester bond [18]. It is generally believed that 2,3’-cGAMP has a stronger affinity for STING than bacterial cyclic di-GMP does, thus eliciting a more robust response [19]. cGAMP can also be transferred to neighboring cells through gap junctions or membrane transporters [20], triggering immune activation in adjacent cells and thereby amplifying the immune effect.

**Fig. 2:**
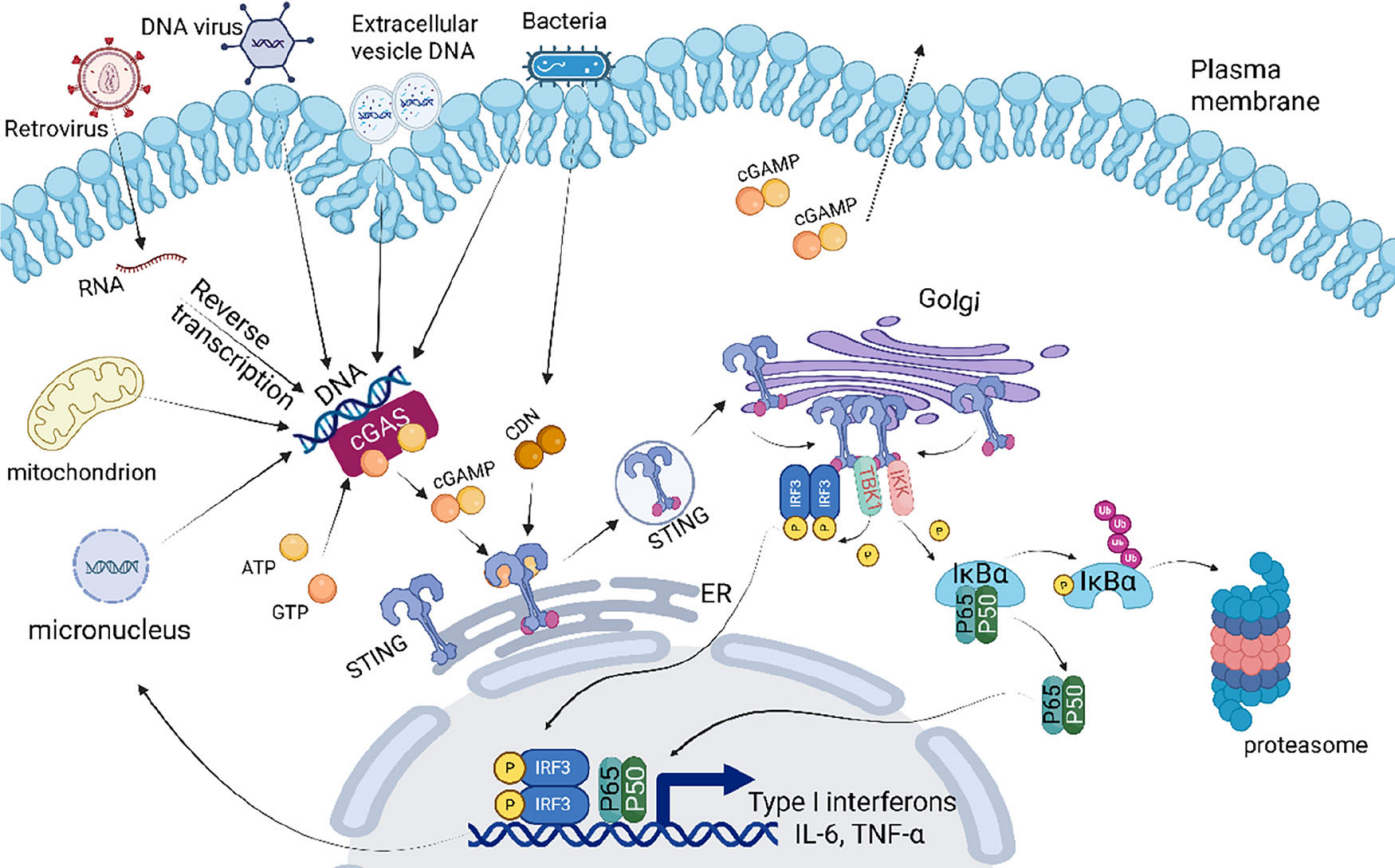
Activation of the cGAS‒STING pathway. The activation of this pathway begins with the exposure of cytosolic dsDNA, which originates from viral genomes, bacterial DNA, tumor cell-released DNA, mitochondrial-derived self-DNA, or self-micronuclear DNA. Upon binding to dsDNA, cGAS catalyzes the synthesis of the cyclic secondary messenger cGAMP from ATP and GTP. The synthesized cGAMP diffuses to the endoplasmic reticulum (ER) membrane and binds to STING. Similarly, bacterial-derived CDNs can also bind to STING. After binding, the STING dimer undergoes a conformational change from a closed to an open state, exposing the phosphorylation sites on its CTD. The activated STING dimer is then translocated from the ER to the Golgi apparatus via vesicular trafficking. At the Golgi membrane, multiple STING dimers further assemble into higher-order oligomers, such as tetramers or polymers. TBK1 is recruited to the STING oligomers, where it undergoes autophosphorylation for activation. The activated TBK1 subsequently phosphorylates the transcription factor IRF3. Phosphorylated IRF3 dimerizes and translocates from the cytoplasm to the nucleus. Then, the IRF3 dimer binds to the promoter regions of IFN-I genes, recruiting transcriptional coactivators to initiate gene transcription. Concurrently, NF-κB exists in an inactive state in the cytoplasm, bound to its inhibitory protein IκB. The phosphorylated STING recruits the IKK complex, which phosphorylates IκB upon activation. The phosphorylated IκB undergoes ubiquitination and is subsequently degraded via the proteasomal pathway. This degradation releases NF-κB, exposing its nuclear localization signal. The activated NF-κB dimer then translocates to the nucleus, where it binds to κB-specific sequences in the promoter regions of target genes, facilitating the recruitment of transcriptional machinery, initiating the expression of pro-inflammatory cytokines and other related genes. Figure created with BioRender.

Upon receiving the cGAMP signal, STING is activated, and the palmitoylation of the Cys88/91 residues in the transmembrane domain plays a crucial role in its activation [21]. Activated STING translocates from the endoplasmic reticulum to the Golgi apparatus via the endoplasmic reticulum–Golgi intermediate compartment (ERGIC), where it recruits and activates TANK-binding kinase 1 (TBK1) and IκB kinase (IKK). Activated TBK1 further phosphorylates the C-terminal domain (CTD) of STING. The phosphorylated CTD recruits IRF3 through its conserved, positively charged phosphate-binding domain. IRF3 bound to the CTD is activated by TBK1 and forms dimers, which then translocate into the nucleus to induce the expression of IFNs such as Interferon-beta (IFN-β) [22]. Additionally, activated IKK phosphorylates the NF-κB inhibitor IκBα, thereby activating NF-κB to induce the expression of pro-inflammatory cytokines such as interleukin-6 (IL-6) and tumor necrosis factor-α (TNF-α) [23] (Fig. 2). To prevent the sustained activation of STING and excessive inflammatory responses, several negative feedback regulatory mechanisms exist within cells. One of these is that TBK1, following pathway activation, can phosphorylate p62/SQSTM1, which directs STING to the autophagosome for degradation [24]. Another mechanism involves cGAMP triggering the dissociation of ULK1 from its repressor AMPK. The dissociated ULK1 can then phosphorylate STING at S366, thereby inhibiting the function of IRF3 [25]. Additionally, the E3 ubiquitin ligase TRIM13 can interact with STING to catalyze the K6-linked ubiquitination of K19 on STING, resulting in its slow exit from the endoplasmic reticulum and degradation [26]. Notably, another member of the E3 ubiquitin ligase family, RNF144A, promotes K6-linked ubiquitination of K236 on STING, which increases the translocation of STING from the endoplasmic reticulum to the Golgi apparatus and thus plays a positive role in the innate immune response [27]. MUL1 catalyzes the ubiquitination of STING at K224, which is essential for the activation of IRF3 [28]. Therefore, the ubiquitination of different lysine residues on STING leads to distinct regulatory outcomes. In addition to receiving signals from cGAS, STING can also be activated in a non-cGAS-dependent manner following viral membrane fusion [29], during the early stages of DNA damage induced by etoposide [30], and upon binding to the RNA recognition ligands RIG-I and MDA5 [31]. In the latter two cases, STING functions in a non-translocated and non-phosphorylated manner.

## How do cells avoid the self-activation of cGAS?

**Fig. 3:**
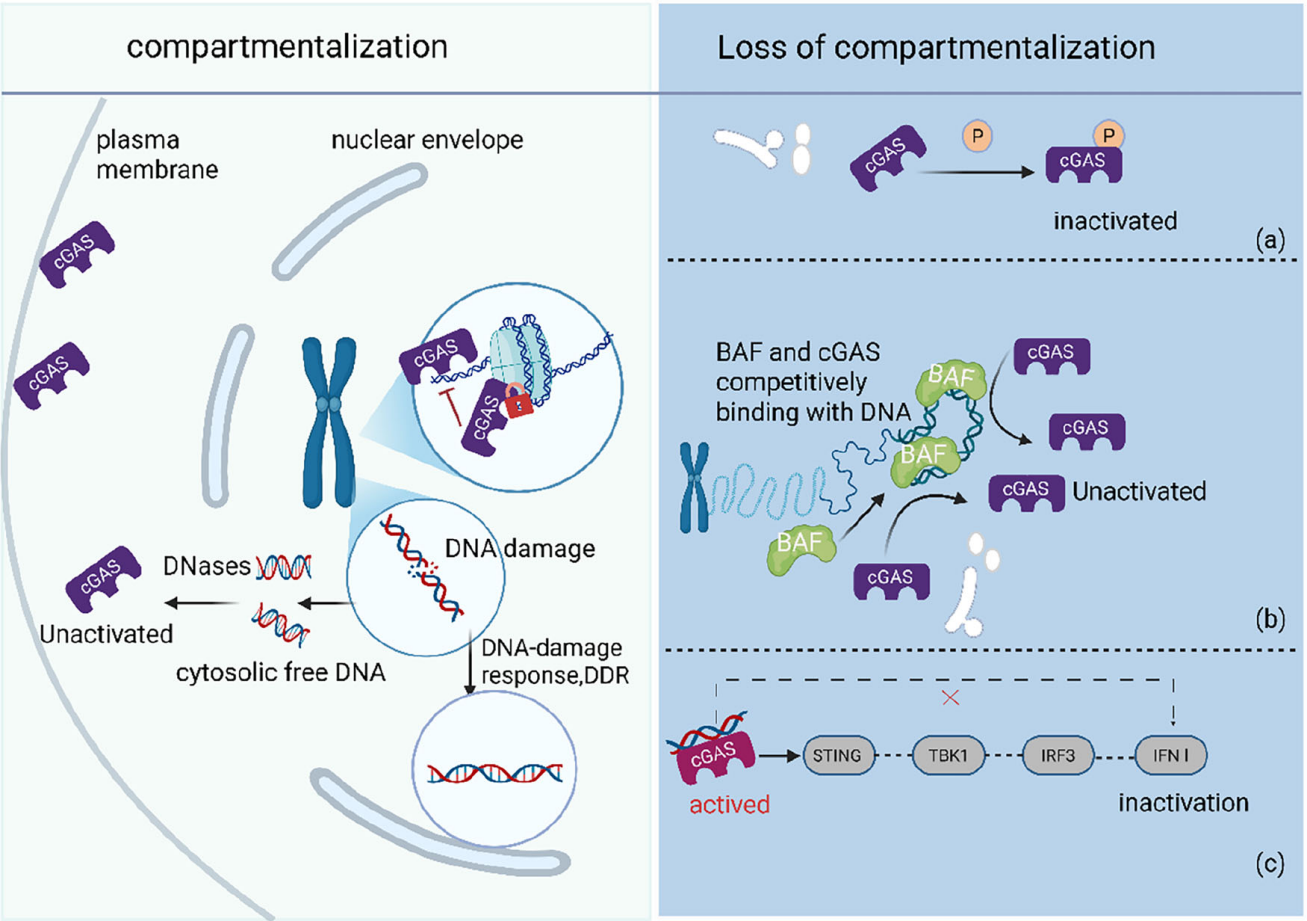
The mechanisms of cGAS preventing self-activation. Normal cells employ multiple mechanisms to prevent inappropriate activation of the cGAS-STING pathway by self-DNA. Left (interphase): nuclear and mitochondrial membranes physically separate genomic DNA from cGAS; quiescent cGAS is tethered to the plasma membrane via its N-terminal domain, distancing it from nuclear DNA. DNA damage promptly triggers the DDR to repair breaks and limit cytosolic DNA leakage; any residual fragments are continuously degraded by cytosolic DNases, keeping DNA levels below the activation threshold. Nuclear cGAS is bound to histones, occluding its DNA-binding surface and remaining catalytically silent. Right (mitosis): after nuclear-envelope breakdown, additional blocks operate. **a** cGAS undergoes inhibitory autophosphorylation. **b** The chromatin-binding protein BAF rapidly occupies DNA, competitively inhibiting cGAS binding. **c** Even if cGAS accidentally engages DNA, the TBK1–IRF3 axis is silenced, preventing signal propagation. Multiple safeguards act in concert to minimize the risk of accidental cGAS-STING activation by host DNA. Figure created with BioRender.

cGAS can sense double-stranded DNA in a length-dependent but sequence-independent manner. In addition to exogenous DNA, the leakage of intracellular self-DNA can also activate this pathway. In our view, cells physically separate cGAS and nuclear DNA through compartmentalization to avoid self-activation. However, even in normal cells undergoing mitosis, the nuclear envelope disassembles, eliminating the barrier between cGAS and nuclear DNA. How do cells avoid self-activation of the cGAS pathway at this time? Moreover, research has shown that cGAS, which is primarily known as an antiviral DNA sensor, is also located in the nucleus and not just in the cytoplasm. What is the rationale and significance of this localization? Furthermore, even during normal cell division, there may be some errors in mitosis or abnormal leakage of DNA from the nucleus. How do cells avoid the abnormal activation of cGAS in such cases? Figure 3 summarizes the strategies employed by cGAS-STING to prevent its own activation. Research and discussion of these questions will help us further understand the role of this pathway in cells and provide another perspective on the occurrence and development of tumors caused by abnormalities in the cGAS/STING pathway.

### Physical sequestration

Under steady-state conditions, intracellular membranes compartmentalize the cell, confining self-DNA to defined organelles such as the nucleus and mitochondria and thereby physically separating it from the cytosolic DNA sensor cGAS [32]. In addition, cGAS is not a freely diffusing cytosolic protein poised for ligand binding; instead, it is tethered to the plasma membrane through an N-terminal domain that interacts with phosphatidylinositol-4,5-bisphosphate (PI(4,5)P₂) [6]. Upon DNA transfection, cGAS dissociates from PI(4,5)P₂, redistributes into the cytosol, and undergoes liquid–liquid phase separation to initiate signal transduction [6]. This membrane pre-localization minimizes accidental encounters between cGAS and self-DNA in resting cells, providing an elegant safeguard against aberrant autoactivation.

### Maintaining self-DNA below the immunostimulatory threshold

#### The DNA damage response (DDR) reduces the generation of cytosolic DNA

Cellular DNA is constantly threatened by exogenous and endogenous factors, such as ionizing radiation, chemical drugs, and reactive oxygen species (ROS) produced by cellular metabolism. Therefore, DNA within normal cells can be damaged when exposed to these threats or when spontaneous replication errors such as incorrect incorporation of dNTPs, base substitution or deletions occur, leading to base mismatches, single-strand breaks (SSBs), or double-strand breaks (DSBs). If not repaired in a timely manner, the continuous accumulation of DNA damage can lead to genomic instability and hinder the stable transmission of genetic material.

To counteract these damages, cells have evolved various mechanisms for repairing DNA damage, including mismatch repair, base excision repair, nucleotide excision repair, direct repair, homologous recombination repair, and non-homologous end joining repair [33]. These repair mechanisms are achieved through the precise regulation of various enzyme activities, such as those of nucleases, helicases, polymerases, topoisomerases, phosphatases, etc., by cells in response to different types of damage. After DNA damage occurs, the cell initiates a series of signal transductions to participate in DNA repair, which is known as DDR. DNA damage sensors, including the phosphatidylinositol 3-kinase-like protein kinase (PIKK) family members ataxia-telangiectasia mutated (ATM) and rad3-related (ATR), and DNA-dependent protein kinase (DNA-PK) and the poly(ADP)ribose polymerase (PARP) family members PARP1/2, are activated upon sensing DNA damage and recruiting downstream effector molecules such as checkpoint kinase 1/2 (CHK1/2), cellular tumor antigen p53 (p53), H2A histone family member X (H2AX), and breast cancer type 1 susceptibility protein (BRCA1) to participate in DNA repair [34]. The ATR-CHK2 pathway, which has been studied mainly for SSB damage, and the ATM-CHK1 pathway, which has been studied mainly for DSB damage [34], are currently the most extensively researched pathways. Once these signaling pathways are activated, the cell cycle is halted to provide time for DNA repair before the normal cycle is resumed. If the damage is too severe to be repaired, the cell will enter pathways of senescence or apoptosis.

However, when DNA damage exceeds DNA repair capacity or when there are defects in DNA damage repair mechanisms, cells are prone to chromosomal segregation errors, leading to the formation of micronuclei. During the S phase of cell division, the micronuclear membrane ruptures, causing nuclear DNA to be expelled and accumulate in the cytoplasm. Defects in the DDR, including hereditary mutations or epigenetic modifications of DDR-related genes, have been proven to be associated with the occurrence of various diseases [35]. Studies have shown that inactivation of both homologous recombination genes, such as BRCA1/2 [36, 37] and the DNA damage checkpoint gene ATM [38], can activate the cGAS/STING pathway through DDR defect-mediated accumulation of cytoplasmic DNA. Additionally, it has been found that stimulation by DNA-damaging agents can lead to dephosphorylation of the Y215 site of cGAS, thereby promoting the translocation of cytoplasmic cGAS to the nucleus. Nuclear cGAS interacts with PARP1 to inhibit homologous recombination, thus promoting tumorigenesis [39]. Moreover, Src homology 2 (SH2) domain-containing protein tyrosine phosphatase-2 (SHP2) also interacts with PARP1 to inhibit non-homologous end joining (NHEJ), exacerbating genomic instability and activating cGAS [40]. Treatment with lovastatin to activate SHP2 and inhibit PARP1-mediated DNA repair can increase the chemosensitivity of colon cancer. Therefore, defects in the DDR can significantly promote genomic instability, leading to tumorigenesis, and can also indirectly result in the activation of the cGAS‒STING pathway because of the accumulation of cytoplasmic DNA.

#### DNases prevent the accumulation of cytosolic DNA

Under normal circumstances, there is still a certain amount of DNA in the cytoplasm, which may result from DNA damage caused by external physical and chemical factors, metabolic stress, DDR defects, or chromosome segregation errors. To counteract the effects of cytosolic DNA caused by these intra- and extracellular factors, cells possess various DNases that can clear cytosolic DNA as much as possible to ensure normal cell survival. Multiple DNases, including DNase I, DNase II, and TREX1 (DNase III), have been identified in cells and can effectively degrade double-stranded DNA [41]. Among them, DNase I is located mainly in the serum to clear chromosomal DNA released by dead cells [42]. DNase II is located in the lysosomal lumen, degrading nuclear DNA engulfed by phagocytic cells and clearing mitochondrial DNA (mtDNA) or micronuclei produced by toxic stress in non-phagocytic cells [43]. TREX1 is anchored to the endoplasmic reticulum, but its exonuclease domain is preserved in the cytoplasm, effectively clearing cytosolic DNA [43]. Their presence can effectively prevent the occurrence of chronic inflammation and autoimmune diseases [44, 45].

On the basis of the different localizations and functions of these enzymes, TREX1 plays a more prominent role in degrading cytosolic DNA. TREX1 can degrade nuclear DNA when micronuclei rupture, thereby inhibiting the activation of cGAS at micronuclei [46]. Additionally, studies have shown that after DNA damage, TREX1 can translocate to the nucleus to participate in DNA repair [47]. However, experiments have also shown that TREX1 can be transferred to the nucleus after nuclear membrane rupture to induce DNA damage [48]. These two seemingly contradictory functions of TREX1 may be related to the degree of DNA damage. When nuclear DNA suffers irreparable damage, TREX1 participates in causing DNA damage, inducing the production of cellular inflammatory factors and promoting cell senescence and autophagy. In cells that normally need to activate the cGAS pathway to exert immune functions, cGAS‒DNA phase separation is used to protect the cGAS‒DNA complex, limiting the DNA-degrading activity of TREX1 to the outer shell of the droplet, resisting the negative regulation of the cGAS pathway by TREX1, and promoting immune activation in cells [49]. p53 induces TREX1 degradation via ubiquitination, causing cytoplasmic DNA accumulation, which activates the cGAS-STING pathway and triggers IFN-I production, exerting antitumor effects [50]. Although cells can activate cGAS to perform immune functions by physically isolating TREX1 from cytosolic DNA or downregulating TREX1 activity, the overactivation of exonuclease 1 can also lead to excessive DNA degradation, increasing DNA damage and promoting chromosomal instability. Therefore, the functional balance of DNases in cells is crucial for normal cell life.

### Strategies to prevent cGAS self-activation during mitosis

#### Nucleosome histones competitively bind cGAS with DNA

The cGAS-STING pathway was initially identified as an antiviral pathway; thus, it is generally believed that, as a DNA sensor, cGAS exists only in the cytoplasm to detect viral DNA and activate the innate immune response of the organism. However, an increasing number of studies have shown that cGAS also has nuclear localization in normal cells [7], in addition to its presence in the cell membrane [6]. The specific localization mechanisms and dynamic changes in cGAS still need to be further clarified.

Gentili [51] et al. reported that the localization of cGAS is related to its N-terminal domain. However, they argued that cGAS is a nuclear protein and that different segments of the N-terminal domain control its localization in the cytoplasm or nucleus. They also confirmed biochemically and microscopically that cGAS does exist in the nuclei of interphase cells, possibly due to the rupture of the nuclear membrane during mitosis and the subsequent encapsulation of cGAS in the nuclei of daughter cells before the formation of new nuclear membranes. Additionally, they reported that cGAS is enriched in centromeric satellites and long interspersed nuclear element (LINE)–DNA repeat sequences, but nuclear cGAS is highly unresponsive to self-DNA, suggesting that there may be some unknown mechanisms regulating the activity of nuclear DNA toward cGAS. The specific role of the N-terminal domain of cGAS and its contradictory localization are not limited to the above findings. Hannah E. Volkman [10] et al. suggested that cGAS is a nuclear protein, regardless of the cell division phase or activation state, and that transfection with DNA does not cause cGAS translocation. The only common point is that the deletion of the N-terminal domain leads to the nuclear localization of cGAS. Endogenous cGAS is tightly bound to the nucleus with force, and this tether is mediated by intact chromatin rather than specific amino acids of cGAS. These differences may be related to the cell type and the specific conditions in which the cells are situated.

Research on how nuclear cGAS is inhibited has gradually increased, revealing that the key to inhibiting its activity is the competitive binding of nuclear cGAS to nucleosomes. cGAS uses two conserved arginines to anchor onto the acidic patch of the nucleosome histone H2A-H2B heterodimer, spatially blocking the binding of nuclear DNA to cGAS and preventing the oligomerization of nuclear cGAS into an active state, thereby reducing the self-activation of cGAS by nuclear DNA [9, 52, 53]. During interphase, nuclear cGAS is inhibited through spatial separation, but how is this inhibition achieved during the dynamic process of partial decondensation of nucleosomes and DNA during mitosis? BAF, a chromatin-binding protein, plays an important role in the formation of the nuclear envelope at the end of mitosis. BAF can competitively bind to dsDNA, thereby inhibiting the binding of cGAS to nuclear DNA [54]. In summary, in normal cells, nuclear cGAS is “locked” to prevent excessive inflammation. When activation is needed, a “key” is needed to unlock and release cGAS. Research has found that the protein meiotic recombination 11 homolog (MRE11), which can recognize broken DNA fragments, may act as the “key.” It can release cGAS from histones upon recognition and binding to broken DNA [55].

The specific significance of nuclear cGAS remains to be elucidated, but it can recruit protein arginine methyltransferase 5 (PRMT5) into the nucleus, catalyzing the symmetric dimethylation of histone H3 arginine 2 at the promoters of *Ifnb* and *Ifna4*, promoting the recruitment of IRF3, and enhancing the expression of IFN-Ⅰ, which is important for combating RNA/DNA viral infections [56]. Additionally, nuclear cGAS maintains genome integrity and stability through multiple pathways [57, 58], and from an evolutionary perspective, this finding also indicates that cGAS may have played a key role in other biological processes before acting as a cytoplasmic DNA sensor. More research is needed in the future to explain the significance of nuclear cGAS and its evolutionary changes.

#### cGAS inactivation via phosphorylation

Phosphorylation-induced silencing of cGAS is essential for preventing self-activation during mitosis [59, 60]. Human cGAS Ser305 (corresponding to mouse Ser291) is evolutionarily conserved and becomes hyper-phosphorylated in mitosis through the mitotic kinase complex CDK1–cyclin B1; the CDK1 inhibitor RO-3306 completely abolishes this modification [59]. Overexpression of CDK1–cyclin B1 suppresses cGAS-driven activation of the IFN-β promoter, whereas CDK1 inhibition elevates cGAMP levels in mitotic cells [59]. Thus, the CDK1–cyclin B1 complex phosphorylates and inactivates cGAS during mitosis. Conversely, the phosphatase PP1 dephosphorylates Ser305 as cells exit mitosis, restoring cGAS activity [59]. Independent work in HeLa cells further showed that multiple N-terminal serine and threonine residues of cGAS are hyper-phosphorylated during mitosis [60]; this phosphorylation attenuates both DNA-induced liquid-droplet formation and enzymatic cGAMP synthesis by cGAS.

#### Downstream components are inactivated

Once activated, cGAS amplifies innate immunity through the STING–TBK1–IRF3 axis; therefore, mitotic cells additionally silence downstream adapters to limit self-reactivity. In mitosis, cGAMP-triggered phosphorylation of both MITA/STING and IRF3 is suppressed even though cGAS is engaged, indicating that the pathway is blocked distal to second-messenger synthesis [59]. Although phospho-TBK1 levels rise during mitosis, its activity is scaffold-dependent: STING or MAVS platforms are required to channel active TBK1 toward IRF3 [23, 61]. Deprivation of these adapters decouples “active TBK1” from IRF3 phosphorylation, redirecting TBK1 to alternative mitotic or metabolic substrates. Consistently, IRF3 phosphorylation that does accumulate in mitosis fails to drive IFN-I transcription, representing a transcriptionally inert, non-canonical phosphorylation event [62].

## Antitumor effects of the cGAS-STING pathway

### Safeguarding chromosomal stability

Studies have revealed that the cGAS‒STING pathway can play a direct role in maintaining chromosomal stability to prevent tumorigenesis. For instance, TBK1 regulates chromosome segregation during mitosis by interacting with Cep170 and NuMA, influencing microtubule stability and spindle assembly [61]. cGAS can act as a decelerator and stabilizer of replication forks independent of cGAMP and STING. Loss of cGAS leads to uncontrolled DNA replication, excessive proliferation, and genomic instability [58]. STING regulates cell proliferation by modulating p21 activation through NF-κB and p53. Deletion of STING results in premature activation of cyclin-dependent kinase 1 (CDK1), leading to early S phase entry and mitosis, and increased chromosomal instability [63]. Additionally, in various cancer cell lines (HeLa and U2OS) facing nocodazole-induced mitotic arrest, low expression of cGAS induces chromosome segregation errors and micronucleus formation, which can be partially rescued by replenishing cGAMP to restore proper chromosome segregation [64]. Further studies suggest that the cGAS/STING/TBK1/IRF3 pathway is essential for maintaining chromosomal stability and preventing the formation of micronuclei due to chromosome segregation errors. Mechanistically, this pathway can increase p21 levels to prevent premature G2/M transition, preventing cells with damaged DNA or incomplete genomes from entering mitosis, thereby preventing chromosome segregation errors [64]. These results indicate that the cGAS‒STING pathway can maintain chromosomal stability by regulating the cell cycle. Therefore, loss of cGAS-STING signaling in cells may lead to tumorigenesis in multiple ways, including the formation of chromosomal instability, disruption of cell cycle regulation, and the absence of host immune pro-inflammatory responses.

### Promoting the senescence and death of precancerous cells

Cellular senescence is a normal process triggered by various stresses and is characterized by permanent cell cycle arrest [65]. Senescent cells secrete a range of cytokines, inflammatory factors, and proteases, collectively known as the “senescence-associated secretory phenotype” (SASP). These factors not only promote senescence in neighboring cells but also attract immune cells to clear senescent cells [8, 66]. During tumorigenesis, precancerous senescent cells secrete factors that are subject to immune clearance, thereby impeding tumor proliferation and development [67]. Thus, cellular senescence acts as a significant barrier to tumor progression. The cGAS-STING pathway promotes the secretion of cytokines and anti-inflammatory factors, including IL-6, TNF-α, and several chemokines associated with the SASP [68]. Various triggers of cellular senescence, including oncogenic signals, rely on the cGAS‒STING signaling pathway to drive the expression of inflammatory SASP genes. Conversely, cells deficient in cGAS/STING exhibit a reduced SASP after irradiation, impairing the senescence response in a senescence model [68]. Therefore, the activation of cGAS within tumor cells promotes cellular senescence. On the one hand, it halts the proliferation of precancerous cells through permanent cell cycle arrest. On the other hand, it facilitates immune cell-mediated clearance by secreting SASP factors. However, when certain cells evade this senescence checkpoint and continue to proliferate, they experience telomere shortening, leading to a replicative crisis as another barrier. When cells enter a replicative crisis, which is characterized by extremely short telomere fusion, they experience mitotic delay and loss of telomere protection, resulting in cell death. Studies have shown that cells with unprotected telomeres induce autophagy, in which the cGAS‒STING pathway participates, thereby suppressing tumorigenesis [69, 70].

Additionally, research has indicated that cGAS activation can promote tumor suppression by stimulating ZBP1-RIPK3-MLKL-dependent necroptosis, particularly in breast cancer [55]. Thus, activation of the cGAS-STING pathway enables precancerous cells to self-clear through multiple pathways, thereby preventing further tumor development and progression.

### IFN-mediated activation of immune cells

The most crucial antitumor effect of cGAS-STING pathway activation is the production of IFN-Ⅰ. IFN-Ⅰ promotes the maturation of dendritic cells (DCs) and cross-prime adaptive immune CD8 T cells [71, 72]. Additionally, activation of this pathway can maintain the stemness of CD8 T cells by regulating the expression of the transcription factor T cell factor 1 (TCF-1) and inhibiting Ak strain transforming (Akt) activity, thereby enhancing antitumor effects [73]. Therefore, the cGAS‒STING pathway serves as a bridge for initiating both innate and adaptive immunity. Given that the priming of spontaneous tumor antigen-specific T cells depends on IFN-Ⅰ and cross-presenting DCs [74], the cGAS-STING pathway is an essential upstream pathway for IFN-Ⅰ production [75]. In STING-deficient mice, there is a defect in CD8+ T-cell priming and a reduction in tumor-specific CD8+ T-cell infiltration [76]. However, the use of exogenous 2′3′-cGAMP can increase the number of tumor-specific CD8+ T cells [77]. Studies have also shown that, in addition to activating CD8+ T cells through IFN-Ⅰ as a co-stimulatory signal, the intrinsic cGAS‒STING pathway can directly activate the immunoproteasome/MHC class I pathway to activate CD8+ T cells [78]. This finding provides an alternative pathway for CD8+ T-cell activation and offers additional insights for tumor immunotherapy targeting the cGAS-STING pathway. In hepatocellular carcinoma, cGAS-STING pathway activation due to DNA repair deficiency can reprogram tumor-associated macrophages in the tumor microenvironment to the M1 type, thereby recruiting infiltrating T cells into the tumor [36]. In addition to the antitumor effects of the DC‒CD8+ T-cell axis, the DC‒NK cell axis, when activated by the cGAS‒STING pathway, can also exert strong tumor-killing effects [79]. Moreover, tumor-derived cGAMP and STING activation in NK cells can promote the expansion of immature TCF-1 NK cells, prolonging antitumor effects [80]. Additionally, Ho reported that STING deficiency in prostate cancer reduces the incidence of tumor cell phagocytosis, suggesting that macrophages also rely on STING to exert antitumor effects [81]. In the tumor microenvironment, in addition to immune cells with tumor-killing functions, there are also cells that contribute to tumor progression, such as regulatory T cells and myeloid-derived suppressor cells (MDSCs). MDSCs primarily target T cells for immune suppression and promote tumor progression by affecting the tumor microenvironment and angiogenesis [82]. STAT3 inhibition or BANF1 inhibition to activate STING signaling has been shown to resist tumor progression by reducing regulatory T cells and MDSCs [83, 84]. cGAMP enhances STING activity in tumor-bearing mice and then activates CD8+ T cells to produce interferon-γ, thereby reducing the number of MDSCs [85]. Therefore, cGAS-STING pathway signaling can activate various immune cells, including DCs, NK cells, macrophages, and CD8+ T cells, and maintain their stemness to exert more durable antitumor effects. It can also reduce the number of immunosuppressive cells, such as regulatory T cells and MDSCs, to prevent tumor progression. Although activation of this pathway can lead to the development of an immunosuppressive microenvironment [86], this needs to be analyzed specifically in different types of tumors and at different stages of development and should not overshadow the powerful antitumor immune response exerted by this pathway.

### Transfer of tumor DNA or cGAMP to neighboring cells to activate cGAS

Since the discovery that tumor-derived DNA can activate cGAS/STING-mediated DC activation and infiltration [75], the mechanisms of tumor DNA transfer to the cytoplasm of DCs have attracted increasing interest. Studies have shown that exosomes can carry various tumor antigens, including proteins, RNA, and miRNA. Similarly, tumor cells treated with irradiation or topotecan (TPT) (a topoisomerase I inhibitor) can produce exosomes containing dsDNA that enter the cytoplasm of DCs, thereby activating cGAS-STING [87, 88]. In addition to tumor-derived dsDNA, cGAMP, the intracellular second messenger produced after cGAS activation, has been found to play a secondary role as an immune mediator. Tumor cell-derived cGAMP can be transferred out of the cell and into immune cells through various pathways, including gap junction transfer and protein transport [89]. Initially, cGAMP was found to be transferred into neighboring cells through gap junctions in virus-infected cells to enhance antiviral responses [90], and later, in brain tumors, gap junctions were found to transfer cGAMP to astrocytes [91]. ABCC1, a member of the ATP-binding cassette (ABC) transporter family, was found to mediate cGAMP export, as inhibiting ABCC1 suppresses cGAMP output [92]. Several members of the solute carrier (SLC) family have been confirmed to be involved in cGAMP import, with solute carrier family 19 member 1 (SLC19A1) identified as the importer of cGAMP in monocyte-derived cells [93] and solute carrier family 46 member 2 (SLC46A2) considered the importer of tumor-derived cGAMP into macrophages and CD14 monocytes [94]. Additionally, the P2X purinoceptor 7 receptor (P2X7R), a member of the P2X family, is a unidirectional importer of cGAMP [95]. The human host defense peptide LL-37 has also been found to act as a cGAMP transporter, specifically by binding to cGAMP and effectively delivering it to target cells [96]. Lastly, the volume-regulated anion channel (VRAC), particularly the leucine-rich repeat-containing 8C (LRRC8C) channel, has been identified as a bidirectional transporter of cGAMP that mediates cGAMP transfer in T cells, leading to STING and p53 activation [97]. Because cGAMP functions as an immune mediator, it can be transferred from tumor cells to immune cells, thereby enhancing the antitumor immune effects of the cGAS-STING pathway.

Moreover, studies have shown that tumor-derived cGAMP activates the STING signaling pathway in endothelial cells, which promotes immune cell infiltration and exerts tumor-suppressive effects by slightly increasing vascular permeability and upregulating adhesion molecules [98, 99].

## Adaptation of chromosomally unstable tumors to the cGAS-STING pathway

In their quest for rapid proliferation, tumor cells often evade cell cycle checkpoint surveillance, leading to frequent chromosomal missegregation events during mitosis, such as the formation of lagging chromosomes and chromosomal bridges. Multiple rounds of replication result in a significant increase in micronuclei formation within tumor cells. It has been reported that cGAS can detect micronuclear DNA, and the fragile micronuclear membrane is prone to rupture, leading to the accumulation of cytosolic DNA [100]. How do tumor cells reconcile the formation of micronuclei and cytosolic DNA accumulation due to genomic instability with the activation of the cGAS pathway?

As chromosomes become increasingly unstable, tumor cells gradually downregulate downstream interferon signaling to adapt to the chronic activation of cGAS/STING by cytosolic DNA. The presence of cytosolic DNA and cGAS/STING does not necessarily indicate the production of IFN-Ⅰ, as tumor cells can inhibit the production of endogenous IFN-I through various pathways. One possible mechanism involves retaining cGAS/STING activity while inhibiting the IFN-Ⅰ-producing pathway downstream of STING, with other pathways downstream of STING, such as NF-κB activity, remaining unaffected [101]. The p38 MAP-kinase stress pathway is believed to be activated in chromosomally unstable tumors and can negatively regulate STING, thereby inhibiting IFN-Ⅰ signaling. The use of p38 inhibitors has been shown to affect the IFN-Ⅰ-producing pathway downstream of STING without impacting other related pathways. Additionally, specific modulation of the non-canonical NF-κB pathway may counteract the positive effects of IFNs. Studies have shown that in DCs, activation of the non-canonical NF-κB pathway (p52/RelB) leads to competitive binding of RelB with RelA of the canonical pathway to the IFNB promoter, impairing IFN-Ⅰ production [102, 103]. Blocking the activation of the non-canonical NF-κB pathway effectively promotes tumor regression. Moreover, genomically unstable tumors may avoid persistent cytosolic DNA stimulation by losing the interferon gene cluster on chromosome 9p during cell division, thereby coexisting with the cGAS-STING pathway.

Pan-cancer data analysis and research findings indicate that tumor cells rarely inactivate cGAS/STING through genetic mutations [104]. Instead, some tumor cells maintain survival by reducing cGAS/STING expression levels or employing protein loss strategies [105, 106], as studies have shown that the consequences of STING activation are highly dependent on the STING protein expression level [107]. For instance, high methylation and transcriptional repression of the cGAS/STING promoter region have been observed in colorectal cancer, melanoma, and ovarian cancer [105, 106, 108]. In addition to methylation, tumor cells also use other epigenetic modifications and post-translational modifications to suppress endogenous cGAS/STING expression [109]. Slightly different from the above perspective, Li suggested that during tumor progression and metastasis, genomically unstable tumor cells do not rely primarily on large-scale loss of the STING protein [110]. Instead, they reconfigure the downstream pathways after STING activation—avoiding the detrimental effects of pro-inflammatory IFN-I pathways, owing to immune suppression induced by ER stress.

## Oncogenic effects of the cGAS-STING pathway

### Immunosuppression by weakening T and B-cell activity or proliferation

During the development of tongue squamous cell carcinoma, STING expression is upregulated. The increased activation of STING induces the release of immunosuppressive factors, such as interleukin-10 (IL-10), indoleamine 2,3-dioxygenase (IDO), and C-C motif chemokine ligand 22 (CCL22) [111]. An increase in Foxp3 Tregs is associated with elevated CCL22 levels, and CCL22 has been shown to mediate Treg infiltration through CCR4 in esophageal squamous cell carcinoma, promoting tumor immune suppression [112]. IDO, which is highly expressed in various tumors, depletes tryptophan, an essential amino acid for T-cell proliferation and activation, thereby inhibiting T-cell activation. The STING-IFNI pathway induces IDO activity, promoting LLC growth, whereas STING-deficient LLC cells eliminate local IDO induction [113]. Thus, the STING-IFNI pathway also suppresses T-cell proliferation and activation by inducing IDO activity, leading to local tumor immune tolerance.

The activation of STING and IFN-Ⅰ or other pro-inflammatory cytokines can induce antiviral cell signaling pathways, such as the JAK-STAT1/2 pathway and the IL-6-JAK-STAT3 pathway, through autocrine or paracrine mechanisms [83]. However, studies have shown that STAT3 negatively regulates the activation of STAT1-dependent inflammatory genes and IFNI-mediated antiviral responses but is associated with the secretion of immunosuppressive cytokines and plays a role in tumor proliferation, survival, and metastasis [114]. The combined use of STAT3 inhibitors and STING activators effectively reduces Treg and MDSC infiltration while increasing the accumulation of CD8+ T cells [83]. Therefore, tumor cells may promote immune suppression through IL-6-mediated STAT3 activation. STING activation may also induce ER stress and inhibit mTORC1 signaling, leading to effective growth inhibition of CD4+ T cells [115].

The activation of the STING pathway in T cells themselves appears to have contradictory effects on T-cell-mediated tumor killing. For example, compared with WT mice, T-cell-specific STING-deficient mice exhibit slower tumor growth after HPV infection, with significantly fewer tumor-infiltrating FOXP3 cells and more CD8+ T cells. Studies have shown that T cells, especially naïve T cells, rely on intrinsic STING pathway activation to mediate the phosphorylation of the transcription factors SMAD3 and STAT5, thereby promoting FOXP3 transcription and leading to the expansion of iTregs [116]. Thus, activation of the intrinsic STING pathway in T cells may be involved in regulating the differentiation and expansion of iTregs, resulting in immune suppression. More surprisingly, in contrast to the classic antiviral role of STING, the activation of STING within T cells can lead to T-cell death. In an adoptive T-cell tumor control model, compared with T cells with interferon-deficient STING and WT T cells, STING-deficient T cells better control tumors and experience less death [117]. It is inferred that the activation of STING within T cells mediates non-IFN Ⅰ-dependent T-cell death. Other experiments have revealed that endogenous STING activation in T cells has antiproliferative effects. This activity is unrelated to STING’s TBK1 and IRF3 recruitment activity and IFN-I but is associated with the C-terminal domain of STING, which activates NF-κB [118].

Compared with the more extensively studied T cells, the role of B cells in tumor immunity is also emerging. In addition to the well-known positive antitumor functions of antigen presentation and antibody production, regulatory B cells play a tumor-promoting role in immune suppression and immune tolerance. Studies have shown that the activation of STING in B cells leads to the expansion of regulatory B cells through IRF3-dependent but IFN-I-independent activation of IL-35 production. B cells lacking STING signaling or blocking IL-35 effectively control tumor growth. Further findings indicate that the STING‒IL35 axis in B cells inhibits NK cell proliferation. Immunotherapy with STING agonists is somewhat limited by B-cell-derived IL-35-mediated NK cell suppression, and the combination of IL-35 inhibitors and STING agonists enhances the antitumor effects of NK cells [119]. Therefore, the activation of intrinsic cGAS/STING signaling in immune cells, especially T and B cells, may promote tumor immune suppression. The specific mechanisms need further exploration to effectively improve resistance to cGAS/STING-targeted immunotherapy.

### Immunosuppression through the upregulation of PD-L1

The programmed cell death protein 1 (PD-1) gene was initially discovered by Ishida in studies of mouse T-cell hybridomas undergoing apoptosis and was named PD-1 because of its ability to promote cell apoptosis [120]. The interaction between PD-1 and its ligands programmed death-ligand 1/2 (PD-L1/2) and their role in regulating T-cell activity were subsequently elucidated [121]. As an immunosuppressive molecule, PD-1 promotes the apoptosis of antigen-specific T cells, preventing the occurrence of autoimmune diseases. Over the following decades, PD-1/PD-L1 has become a hot topic in tumor development and immunotherapy. This is because PD-L1 is overexpressed in various tumor cells to inhibit the cytotoxicity of T cells, thereby facilitating immune evasion. Therefore, PD-L1 antibodies have become a popular pathway for tumor immunotherapy [122].

After the cGAS-STING pathway is activated in tumors due to defects in the DDR or irradiation, the paradox of a lack of T lymphocyte-mediated cytotoxicity has been observed in various types of tumors. Although the cGAS-STING pathway mediates immune cell infiltration, it also induces the upregulation of PD-L1 expression in tumor cells, thereby suppressing the cytotoxicity of antigen-specific T cells and achieving immune evasion by tumor cells [36, 37, 123, 124]. After combined PD-L1 antibody treatment, infiltrating T lymphocytes effectively kill tumors and induce tumor regression [36]. The mechanism was explored in hepatocellular carcinoma, where cGAS/STING activation induces the upregulation of PD-L1 expression through IFN-I-mediated STAT1 [36]. In mesenchymal cells activated by irradiation, cGAS/STING activation induces the secretion of CCL5, which has been shown to induce the overexpression of PD-L1 in breast cancer cells [123, 125]. In addition to the IRF3-IFNI pathway, the NF-κB pathway is also a downstream pathway after STING activation. Studies have shown that NF-κB signaling activation upregulates PD-L1, which has been observed in T-cell lymphoma [126], ovarian cancer [127], non-small cell lung cancer [128], and cervical cancer [129]. Therefore, when the cGAS-STING pathway is targeted for immunotherapy, it is necessary to consider the immunosuppression caused by increased PD-L1 expression.

### Oncogenic effects through activation of the non-canonical NF-κB pathway

As discovered in 1986, NF-κB comprises five family members: RelA (p65), RelB, c-Rel, NF-κB1 (p50 and its precursor p105), and NF-κB2 (p52 and its precursor p100) in mammalian cells. These proteins form homodimers or heterodimers that translocate to the nucleus and bind to the promoter regions of target genes to influence gene transcription. The shared Rel homology domain (RHD) of these proteins is crucial for DNA binding and dimerization. Additionally, RelA (p65), RelB, and c-Rel possess transactivation domains (TADs), and any dimer formed with these members will activate gene transcription. In contrast, p50 and p52 lack TADs, and their dimers act as transcriptional repressors when bound to specific regions of target gene promoters. In resting cells, these dimers typically bind with IκB proteins. Characterized by ankyrin repeats, IκB proteins bind to the DNA-binding domain, thereby inhibiting NF-κB activity while ensuring its stable presence in the cytoplasm. However, the precursors of p50 and p52, p105 and p100, respectively, contain ankyrin repeats themselves, thus exerting self-inhibition. They are only activated after being processed into mature forms through cleavage.

The NF-κB pathway is divided into canonical and non-canonical pathways. In the canonical pathway, typical stimuli such as TNFα, lipopolysaccharide (LPS), and interleukin-1β (IL-1β) interact with tumor necrosis factor receptor (TNFR), Toll-like receptor (TLR), and interleukin-1 receptor (IL-1R), leading to the activation of the IKK complex. Activated IKK primarily phosphorylates IκBα through IKK2, subsequently resulting in ubiquitin‒proteasome degradation of the IκB complex. The non-canonical pathway, on the other hand, originates from different receptors, including B-cell activation factor receptor (BAFFR), lymphotoxin β-receptor (LTβR), and CD40. These receptors cause the activation of NF-κB-inducing kinase (NIK), which leads to the phosphorylation and activation of IKK1, thereby inducing the phosphorylation and ubiquitination of p100. p100 is then partially degraded and processed into p52. Since p100 often forms dimers with RelB, activation of the non-canonical pathway typically results in the nuclear translocation of p52-RelB dimers to exert transcriptional activity. The role of NF-κB in tumors is complex and multifaceted [130].

Studies have shown that cGAS/STING signaling can lead to the activation of both the canonical and non-canonical NF-κB pathways. However, during the malignant progression of tumors, the upregulation of the non-canonical NF-κB pathway activated by cGAS/STING plays a more significant role. It has long been demonstrated that activation of the non-canonical NF-κB pathway is advantageous for tumor invasion, migration, and immune evasion [131–134]. Compared with primary tumors, metastatic tumors exhibit higher chromosomal instability (CIN), leading to increased cytosolic DNA accumulation and subsequent activation of the cGAS-STING pathway. Investigations into the underlying mechanisms revealed that in metastatic tumors, activation of the cGAS‒STING pathway results in the upregulation of the non-canonical NF-κB pathway, thereby promoting metastasis [100]. Moreover, tumors with high CIN rely on the activation of the cGAS-STING-STAT3 and non-canonical NF-κB pathways to prevent cell death mediated by STAT1 and ASK–JNK [135].

### Oncogenic effects based on the hydrolysis of cGAMP

cGAMP, as the second messenger produced after cGAS activation, functions not only in intracellular signal transduction but also, as studies have reported, can be exported and imported through transporters on the cell membrane or gap junctions to activate STING activity in host cells within the tumor microenvironment, thereby amplifying the antitumor effect [136–138]. Therefore, cGAMP can be regarded as an immune mediator that is produced intracellularly and released extracellularly to signal to distant cells.

To maintain the balance of cGAMP in the microenvironment, cells secrete hydrolases to degrade extracellular cGAMP. In 2014, the first cGAMP hydrolase was identified: the ectonucleotide pyrophosphatase/phosphodiesterase 1 (ENPP1) [139]. In addition to ENPP1, its homolog ectonucleotide pyrophosphatase/phosphodiesterase 3 (ENPP3) was also identified as a cGAMP hydrolase [140]. Both can be expressed under steady-state conditions and have different tissue expression patterns, representing two non-redundant hydrolases. Unlike the above two stably expressed enzymes, sphingomyelin phosphodiesterase acid-like 3A (SMPDL3A) was identified as a cGAMP-degrading enzyme activated by liver X receptor1 (LXR1) [141].

**Fig. 4:**
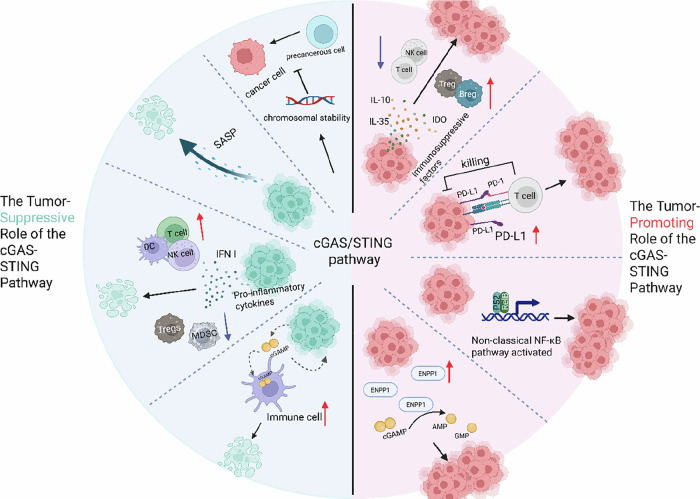
Dual roles of cGAS-STING pathway activation in cancer. Antitumor effects:1. cGAS/STING helps maintain chromosomal stability and thereby prevents tumorigenesis. 2. STING activation induces tumor cell senescence via SASP production and promotes apoptosis. 3. Secreted IFNs and chemokines (e.g., CXCL10) enhance DC maturation, NK/CTL-mediated tumor killing, T-cell activity and suppress immunosuppressive Tregs activity. 4. The paracrine signaling of cGAMP activates the STING pathway in neighboring immune cells, establishing a cascade amplification of immune responses. Oncogenic effects: 1. Enhanced secretion of TGF-β and IL-10 amplifies the immunosuppressive activity of MDSCs and Tregs, while concurrently inhibiting the activity of T and B lymphocytes. 2. Upregulation of PD-L1 on tumor cells inhibits T cell function via PD-1/PD-L1 axis. 3. Activating non-canonical NF-κB signaling drives tumor invasion and metastasis. 4. Upregulation of the hydrolase ENPP1 accelerates cGAMP degradation, impairing antitumor immunity. Figure created with BioRender.

As one of the hallmarks of tumors, genomic instability constantly exposes dsDNA in micronuclei, leading to the activation of cGAS/STING signaling. To adapt to the consequences of this feature, tumor cells must address the issue of cGAMP leakage into the extracellular space and its uptake by other cells. One method employed by tumor cells is to increase ENPP1 expression to hydrolyze extracellular cGAMP, thereby inhibiting the activation of antitumor STING signaling in the tumor microenvironment and controlling tumor metastasis [142]. This study revealed that ENPP1 levels gradually increase in metastatic tumors compared with those in primary tumors. Tumor-derived cGAMP is degraded by ENPP1 to prevent the activation of STING signaling in host cells. Additionally, cGAMP is broken down into adenosine outside the cell, and binding to adenosine receptors on tumor and immune cells helps promote tumor metastasis and immune suppression [139]. In breast cancer, ENPP1 has also been found to act as an immune checkpoint, and targeting ENPP1 can enhance the patient response to PD-L1 immunotherapy [143] (Fig. 4).

## cGAS/STING and immune cell-specific effects

The immune cell-mediated killing is the core execution step of antitumor immunity. The previously mentioned dual roles both involve the participation of immune cells. The cGAS-STING pathway plays complex and distinct regulatory roles in different immune cell subsets—including dendritic cells essential for antigen presentation, CD8⁺ T cells and NK cells responsible for direct killing, as well as highly plastic macrophages and B cells. The specific responses of these cell types collectively shape the regulatory network of the tumor immune microenvironment. To provide a holistic perspective and integrate these key findings, Table 1 summarizes the effector patterns of the cGAS-STING pathway in various immune cells, aiming to clarify which activation modes contribute to building effective antitumor immunity and under which circumstances this process may be impaired.

**Table 1:** Summary of cGAS-STING pathway effects in different immune cells.

Cell type	Antitumor mode of action	Pro-tumor mode of action	Therapeutic implications and potential risks
DCs	Sense tumor DNA/cGAMP, mature via the IRF3/IFN-I pathway, upregulate co-stimulatory molecules, efficiently cross-present antigens, and prime CD8⁺ T cells [183, 184].	-	**Core strategy:** STING agonists targeting DCs are ideal immune “vaccine adjuvants.” **Challenge:** Overcoming low intratumoral DC numbers and inefficient agonist delivery.
CD8⁺ T cells	Moderate activation: May maintain stemness/memory phenotype, enhancing durable antitumor capacity [73].	Excessive endogenous activation: induces non-IFN-I-dependent cell death or exhaustion [107, 118]. promote Treg differentiation under specific conditions.	**Opportunity and risk**: Combination therapies require fine-tuned dosing to avoid direct damage to effector T cells or induction of immunosuppression.
Macrophages	M1 polarization: STING activation promotes polarization to the antitumor M1 phenotype, enhancing phagocytosis, antigen presentation, and pro-inflammatory cytokine secretion [36, 185].	M2 polarization: In certain TMEs (e.g., IL-6-rich), STING may paradoxically promote pro-tumor M2 polarization or PD-L1 expression via STAT3 [83].	**Highly context-dependent:** Requires combination with TME reprogramming (e.g., IL-6/STAT3 blockade) to ensure conversion to a “friend.”
NK cells	Direct activation: Tumor-derived cGAMP activates STING via gap junctions, enhancing NK cell cytotoxicity and IFN-γ production [79, 80].	Indirect suppression: Factors like IL-35, produced by STING-activated B cells, can potently suppress NK function [119].	**Key to combination strategies:** Using STING agonists should consider concurrent blockade of immunosuppressive axes (e.g., IL-35) to unleash NK cell activity.
B cells	Antigen presentation & antibody production: Classical antitumor functions, though less directly impacted by the STING pathway.	Induction of regulatory B cells: STING-IRF3 axis-driven B cells produce IL-35, suppressing NK and CD8⁺ T cell function [119].	**Potential novel target:** Selective inhibition of the STING-IL-35 axis in B cells may relieve their dual suppression of innate and adaptive immunity.

## Targeting the cGAS‒STING pathway for tumor immunotherapy

Currently, most agonists of the cGAS-STING pathway are cGAMP analogs or CDNs that directly activate the STING protein [144, 145]. Although they have shown good efficacy in immune stimulation and antitumor immunity, there are problems such as difficult delivery, susceptibility to enzymatic degradation and clearance, and low bioavailability. Therefore, the development of reliable delivery systems is particularly critical. In addition, compared with normal cells, tumor cells often partially lose the function of DNA replication checkpoints, increasing their sensitivity to DNA damage. Researchers have focused on endogenous activation of the cGAS‒STING pathway, which can not only effectively prevent off-target toxicity but also reduce delivery challenges. On the basis of the signal transduction process of cGAS-STING pathway activation, many immune therapeutic strategies targeting the activation of this pathway have focused mainly on inducing DNA damage in tumor cells or inhibiting their DNA repair to promote the accumulation of cytoplasmic DNA, leading to the endogenous activation of the cGAS-STING pathway in tumor cells and the activation of this pathway in other immune cells.

### cGAS-STING agonists in combinations with radiotherapy

Radiotherapy, one of the main clinical treatments for malignant tumors, exerts its antitumor effect by inducing DNA damage-triggered cell death or by activating the immune system, particularly CD8+ T cells [146]. However, radiotherapy alone may lead to the formation of an immunosuppressive tumor microenvironment and have a limited effect on distant tumors. Therefore, it is necessary to find combination therapies to enhance the immunotherapeutic effects of radiotherapy. DNA damage in tumor cells caused by radiotherapy can activate the cGAS-STING pathway, inducing the release of interferons and the activation of immune cells such as dendritic cells and CD8+ T cells, thereby enhancing the killing effect on tumor cells [77, 147]. In addition, during direct cell‒to-cell contact, cGAS in host DCs can sense the DNA of irradiated tumor cells [77]. Identifying and screening suitable cGAS‒STING pathway agonists in combination with radiotherapy can maximize the therapeutic effect on tumors. It has been found that Mn^2+^ can activate or enhance the activity of the cGAS-STING pathway through multiple pathways, such as directly activating cGAS to catalyze the synthesis of cGAMP or enhancing the sensitivity and catalytic activity of cGAS to dsDNA [148–152]. Considering their stability and delivery, MnO_2_ nanomaterials have been prepared as commonly used agonists for this pathway. With in-depth research, a variety of antitumor immunotherapeutic strategies based on MnO_2_ nanomaterials have been developed. A novel type of lanthanide-doped radiosensitizer-based metal‒phenolic network not only makes tumor cells more sensitive to X-rays but also enhances the activity of the STING pathway with Mn^2+^, improving the therapeutic effect [153]. To reduce off-target effects, a new type of mannose-modified MnO_2_ nanovaccine has been developed to target innate immune cells to activate the STING pathway for tumor therapy [154].

### Combination with immune checkpoint therapy

In recent years, in addition to the “three mainstays” of cancer treatment, immunotherapy has flourished in the field of oncology. Among them, immune checkpoint therapy, which targets the patient’s immune system rather than tumor cells, has also been increasingly developed to induce and enhance the continuous antitumor immune response of T cells. The immune system plays an important role in the surveillance and elimination of cancer, including the recognition and presentation of tumor antigens, the activation of immune cells, the infiltration of T cells, and the final killing and clearance of tumor cells. However, to prevent excessive activation of T cells, “brake molecules,” such as cytotoxic T-lymphocyte-associated protein 4 (CTLA-4) and PD-1, which are inhibitory molecules, have been discovered in sequence and are also known as immune checkpoints. Tumor cells also take advantage of this by upregulating the expression of their ligands to suppress the immune-killing effect of T cells, resulting in immune evasion. Thus, immune checkpoint inhibitor therapy has emerged. The first checkpoint inhibitor, the CTLA-4 antibody ipilimumab, was approved by the US Food and Drug Administration (FDA) for advanced melanoma in 2011. In 2014, the PD-1-blocking antibodies pembrolizumab and nivolumab were approved by the FDA for advanced melanoma [155]. Although immune checkpoint therapy has achieved certain results in cancer treatment, clinical treatment has shown that a single inhibitor is effective for only a few patients.

Wang et al. reported that PD-L1 antibodies exert antitumor effects in wild-type mice but not in cGAS/STING-deficient mice [156]. It is believed that the cGAS-STING pathway can increase the infiltration of leukocytes, including tumor-specific CD8+ T cells, during PD-1-blocking treatment. On the other hand, activation of the cGAS-STING pathway can promote T-cell infiltration but also increase the expression of PD-L1 in tumor cells [36]. Based on the above research, in addition to the use of agonists to activate this pathway during immunotherapy, their combination with PD-1/PD-L1 inhibitors significantly enhances the antitumor immune effect. Wang et al. reported that intramuscular injection of cGAMP combined with PD-L1 antibodies results in better antitumor effects than any single treatment does, inhibits the growth of mouse melanoma, and prolongs the survival of tumor-bearing mice [156]. To overcome the challenges associated with the delivery and degradation of cGAMP, nanomaterials may have better therapeutic prospects. Deng et al. developed MnO_2_ nanomaterials combined with αPD-L1, which enhanced the antitumor immune response and inhibited tumor growth and metastasis by releasing Mn^2+^ to activate the cGAS-STING pathway and exerting immune checkpoint-inhibitory effects on αPDL1 [157]. Huang et al. developed a type of nanogranule targeting liver cancer with a dendrimer shell that contains plasmids encoding IL-2 and PD-L1 siRNA, which can increase the infiltration of immune cells into tumors and the activation of CD8+ T cells and reprogram the immunosuppressive microenvironment [158]. Therefore, considering the activation of the cGAS‒STING pathway to achieve immunotherapy while also considering immune checkpoint inhibition will yield better results. In addition to PD-1/PD-L1 inhibitors, the functions and effects of combining cGAS/STING agonists with other immune checkpoint inhibitors can be explored in the future to find better therapeutic combinations.

### Immune therapy based on nuclear DNA damage

Immune therapeutic strategies based on the induction of DNA damage to activate the cGAS pathway have been explored. Ling et al. designed two PtII complexes (Pt1 and Pt2) that can damage DNA and induce the release of cGAMP, promoting the maturation of dendritic cells and inhibiting tumor growth upon photoactivation [159]. Wang et al. developed a protein-based nanoagonist that combines Mn^2+^ and β-lapachone. β-Lapachone induces immunogenic tumor cell apoptosis, releasing abundant dsDNA into the TME, while Mn^2+^ enhances the sensitivity of cGAS to dsDNA and augments STING signaling to trigger downstream immune-stimulating signals [160]. Common chemotherapeutic drugs also target tumor cell DNA, causing replication errors or hindering repair, leading to cell death. In BRCA-deficient breast cancer, the absence of homologous recombination repair for DSBs means that these cells cannot tolerate the accumulation of DNA damage caused by PARP inhibitors. Therefore, PARP inhibitors are effective in treating BRCA-deficient breast cancer, partly because the DNA damage caused by PARP inhibitors activates the cGAS‒STING pathway, resulting in T-cell infiltration and activation [161]. In addition, the inhibition of proteins related to the DNA damage response, such as ATM [162, 163], CHK2 [164], and topoisomerase [162], can also achieve antitumor immunity through the cGAS-STING pathway.

In addition to causing intracellular DNA damage with certain chemicals, directly assembling exogenous dsDNA that is internalized and released by cells to activate the cGAS pathway has also become a method of tumor immune therapy. Li et al. synthesized CaCO_3_ microparticles templated with dsDNA [165]. After entering the body, the released DNA activates the cGAS pathway in DCs. The results showed that this material significantly inhibited the growth of mouse CT26 and B16F10 tumors in vivo. Since cGAS has no sequence specificity for dsDNA binding, DNA from other sources can also be used to synthesize similar DNA@CaCO3 structures with the same ability to activate innate and adaptive immunity to inhibit tumors.

### Immune therapy targeting mitochondrial DNA

When cellular DNA is damaged by external factors, most nuclear DNA is extensively fragmented to prevent autoimmunity. The association of nuclear DNA with histones in the form of chromatin also restricts its export from the nucleus. However, mtDNA, lacking the protection of histones, is more susceptible to damage and leakage into the cytoplasm, thereby activating the cGAS-STING pathway. The release mechanism of mtDNA may involve the permeabilization of the mitochondrial outer membrane (MOMP), followed by the expulsion of mtDNA from the mitochondrial inner membrane into the cytoplasm through pores mediated by BAX/BAK [166]. Based on this strategy, a nanoplatform that encapsulates Hemin (an efficient iron-containing catalyst), BSO (a GSH biosynthesis inhibitor), and Mn^2+^ (a cGAS-STING activator) [167]. The combined action of Hemin and BSO induces high ROS levels in tumor cells, causing mitochondrial oxidative stress and the release of mtDNA. Mn^2+^ enhances the sensitivity and binding capacity of cGAS to DNA, thereby amplifying the activation effect. In addition, this nanoplatform can effectively trigger ferroptosis in tumor cells, releasing tumor-derived cytoplasmic DNA and activating the cGAS‒STING pathway in DCs. Following the same strategy and adding tumor-targeting functions, Wen et al. developed a nanomaterial containing PpIX, which can cause high levels of ROS under light irradiation and dysregulation of mitochondrial redox homeostasis, leading to mtDNA leakage, and Mn^2+^, which can increase the activity of cGAS-related proteins and assemble hyaluronic acid (HA) on the surface to target CD44 in breast tumor cells [168]. The results showed that this method has a significant effect on the antitumor immune response. Targeted mitochondrial photodynamic therapy has shown initial efficacy in cancer treatment, utilizing the characteristics of photosensitizers (PSs) to produce ROS in the presence of oxygen upon irradiation with light of a specific wavelength to kill cells [169, 170]. Moreover, it is equipped with tumor-targeting antibodies to achieve the purpose of tumor cell death. The targeted mitochondria induce cell death by affecting energy metabolism, apoptotic pathways, and ROS homeostasis, among which the production of ROS involves the release of mtDNA and the activation of the cGAS-STING pathway. Therefore, the activation of the cGAS-STING pathway in immune therapy while targeting mitochondrial photodynamic therapy significantly enhances the effectiveness of tumor treatment. In addition to biosynthetic nanomaterials, plant-derived nanovesicles (PDNVs) have been found to be a mechanism for interaction and communication with mammals. Nanovesicles extracted from artemisinin (ADNV) have been shown to be internalized by tumor-associated macrophages (TAMs), and the contained mtDNA can activate cGAS-related pathways and drive TAMs to shift to an antitumor phenotype to drive tumor regression [171].

### ENPP1 inhibitors

Research has shown that ENPP1 is highly expressed in various tumors, including ovarian cancer, breast cancer, and glioblastoma, and its overexpression leads to strong immune suppression and is associated with poor prognosis [172]. ENPP1 is a cGAMP extracellular hydrolase. Therefore, ENPP1 may also be a target for cancer immunotherapy. Previous studies have shown that after cGAS in tumor cells is activated, it produces cGAMP, which not only activates the STING pathway in the cell itself but also releases cGAMP into the extracellular space to activate the cGAS‒STING pathway in other immune cells [142]. However, in many tumors, tumor cells can regulate the activity of ENPP1 to hydrolyze extracellular cGAMP. On the one hand, this prevents signal transmission to other immune cells, and on the other hand, it promotes their own metastasis through the production of the immunosuppressive molecule adenosine after the hydrolysis of cGAMP [143, 173]. Therefore, the development of inhibitors targeting the ENPP1 molecule has also been proven to effectively inhibit the growth and metastasis of tumors [174] (Table 2).

**Table 2:** ENPP1-targeted inhibitors.

Inhibitor name	Type	Mechanism of action	Preclinical model	Key effects
STF-1623/CM-3163 l [186, 187] (Angarus)	Small-molecule inhibitor	Cell-impermeable, Zn²⁺-chelating ENPP1 inhibitor	Breast cancer model	Reduced locoregional failure, decreased MDSCs/M2-TAMs, enhanced DC/CD8⁺ T/NK cell activity
AVA-NP-695 [188] (Avammune)	Oral small-molecule inhibitor	Cell-permeable, high oral bioavailability	Breast cancer model	Monotherapy-driven tumor volume reduction
TXN10128 [189]	Oral small-molecule inhibitor	Selective ENPP1 inhibition	Colon cancer model	Synergistic tumor growth suppression with anti-PD-L1, increased TILs
POM1 [190] (Sodium polyoxotungstate)	Broad-spectrum ectonucleotidase inhibitor	Nonspecific inhibition of CD39 and ENPP1 at high doses	Pan-cancer models	Reduced metastatic burden, enhanced antitumor immunity
ZX-8177 [191]	Small-molecule inhibitor	Potent and selective ENPP1 inhibition	Murine colon cancer model	Tumor growth inhibition (mono/combination), immune microenvironment remodeling
RBS2418 [192] (Riboscience)	Oral small-molecule inhibitor	Selective ENPP1 inhibition, innate immune activation	Advanced metastatic tumor models	Tolerable monotherapy with antitumor responses
SR-8541A [193]	Oral small-molecule inhibitor	Potent and selective ENPP1 inhibition	advanced metastatic solid tumors	Safe and tolerable monotherapy with antitumor responses
Anti-ENPP1 antibodies [194]	Antibody/ADC/CAR-T	ENPP1-targeted antibodies, antibody‒drug conjugates (ADCs), T-cell engagers, CAR-T cells	ENPP1-expressing tumor cells	Potent cytotoxicity against ENPP1⁺ cells

### Clinical translation status and challenges

Despite the significant attention garnered by the cGAS/STING pathway as a novel immunotherapy target, leading to numerous clinical trials (primarily evaluating STING agonists as summarized in Table 3). Its clinical translation remains precarious and faces multi-layered hurdles that keep it far from a mature therapeutic modality.

**Table 3:** Summary of STING agonists in clinical development.

Drug name	Route	Combination	Indications	Phase/status	NCT number
ADU-S100 (MIW815)	IT	PD-L1 mAb	Solid tumors/lymphoma	I/Terminated	NCT03172936 [195]
ADU-S100 (MIW815)	IT	Pembrolizumab	HNSCC	II/Terminated	NCT03937141 [196]
ADU-S100 (MIW815)	IT	Ipilimumab	Adv./Met. solid tumors/lymphoma	I/Terminated	NCT02675439
Ulevostinag (MK-1454)	IT	Pembrolizumab	Solid tumors/lymphoma	I/Completed	NCT03010176 [197]
Ulevostinag (MK-1454)	IT	Pembrolizumab	HNSCC	II/Completed	NCT04220866 [198]
CRD3874-SI	IT	Monotherapy	AML	I/Active, not recruiting	NCT06626633
GSK3745417	IV	Dostarlimab	Neoplasms	I/Active, not recruiting	NCT03843359
GSK3745417	IV	Monotherapy	AML	I/Terminated	NCT05424380 [199]
SYNB1891	IT	Atezolizumab	Metastatic solid tumors/lymphoma	I/Terminated	NCT04167137 [200]
TAK-676 (Dazostinag)	IV	Pembrolizumab + RT	HNSCC/TNBC/NSCLC	I/Completed	NCT04879849 [201]
TAK-676 (Dazostinag)	IV	Pembrolizumab + platinum+5-fluorouracil	Solid tumors	I/II/Active, not recruiting	NCT04420884 [202]
IMSA-101	IT	ICI/IO	Adult solid tumors	I/II/Completed	NCT04020185 [203]
IMSA-101	IT	ICI	Adult solid tumors (rollover)	I/Active, not recruiting	NCT06026254
IMSA-101	IT	ICI + RT	mRCC/oligoprogressive disease	II/Recruiting	NCT06601296
SNX-281	IV	Pembrolizumab	Advanced solid tumors/lymphoma	I/Terminated	NCT04609579 [204]
exoSTING (CDK-002)	IT	Monotherapy	Advanced solid tumors	I/II/Completed	NCT04592484
ONM-501	IT	Cemiplimab	Advanced solid tumors/lymphoma	I/Recruiting	NCT06022029 [205]
BI 1387446	IT	Ezabenlimab	Advanced solid tumors	I/Completed	NCT04147234 [206]
TAK-500	IV	Pembrolizumab	Metastatic solid tumors	I/II/Terminated	NCT05070247 [207]
BI 1703880	IV	Ezabenlimab	Solid tumors	I/Recruiting	NCT05471856 [208]
SB 11285	IV	Atezolizumab	Advanced solid tumors	I/Completed	NCT04096638 [209]
XMT-2056	IV	Monotherapy	Solid tumors	I/Recruiting	NCT05514717 [210]
BMS-986301	IT	Nivolumab + Ipilimumab	Advanced solid tumors	I/Completed	NCT03956680
E-7766	-	Monotherapy	Lymphoma/advanced solid tumors	Ⅰ/Terminated	NCT04144140 [211]
HG381	IV	Monotherapy	Advanced solid tumor	Ⅰ/Recruiting	NCT04998422
KL340399	-	Monotherapy	Advanced solid tumor	Ⅰ/Completed	NCT05387928
ONO-7914	-	ONO-4538	Solid tumor	Ⅰ/Terminated	NCT06535009

The foremost obstacle is the fundamental tension between delivery and safety. More than half of the agents (e.g., ADU-S100, Ulevostinag) are administered intratumorally, which effectively avoids systemic toxicity and validates local immune activation, but restricts use to accessible solid lesions such as HNSCC and excludes hematologic or diffusely metastatic disease. Conversely, intravenous agonists (e.g., TAK-676, GSK3745417) designed for systemic reach are confronted with dose-limiting, class-related toxicities (CRS, thrombocytopenia), leading to early termination of several programmes (SNX-281, TAK-500). This “locally effective but limited vs. systemically feasible yet unsafe” dilemma constitutes the most stubborn bottleneck.

Second, combination with immune-checkpoint inhibitors has become the default strategy, yet it has not meaningfully increased success probability. The majority of listed regimens pair STING agonists with anti-PD-1/PD-L1 antibodies; nevertheless, terminated trials account for a large proportion of these combinations. Simple mechanistic additivity amplifies immune activation but also expands the risk of immune-related adverse events without widening the therapeutic window. Notably, ADU-S100 plus ipilimumab was halted. Moreover, complex platforms such as ADCs (XMT-2056) and engineered microbial vectors (SYNB1891) exhibit high early failure rates, underscoring additional technological barriers in targeted delivery, manufacturability and in vivo controllability.

Finally, the clinical development path is characterized by high attrition, with phase II acting as a critical graveyard. Although the pipeline appears crowded, advancement is heavily concentrated in phase I. Importantly, every candidate that entered phase II (ADU-S100 in HNSCC, Ulevostinag solid-tumor basket) has been completed or terminated, and none has progressed to a pivotal phase III, implying that demonstration of consistent, clinically meaningful benefit in larger cohorts is presently unattainable.

In summary, future success will hinge less on developing more analogs and more on next-generation, tumor-selective delivery technologies (smart nanomaterials, liposomes, ADCs) that bypass the intratumoral-versus-systemic trade-off, rationally designed combination schedules, and precision patient-stratification tools (STING-pathway activity biomarkers) to convert robust preclinical science into broad clinical benefit.

## Beyond cancer: the double-edged sword role of the cGAS-STING pathway in regenerative medicine

Traditionally, regenerative medicine and cancer therapy have been viewed as opposing fields, with the former aiming to promote tissue repair and reconstruction, and the latter focused on curbing abnormal proliferation. However, in-depth research into the cGAS-STING pathway reveals that both fields share core mechanisms in cellular homeostasis regulation, such as proliferation, migration, survival, and responses to microenvironmental signals. The crucial distinction lies in whether these processes are under precise spatiotemporal control, highlighting the broad pathophysiological significance of this pathway.

In regenerative medicine, the role of the cGAS-STING pathway is also context-dependent—a “double-edged sword.” Sustained or excessive activation of the cGAS-STING pathway has become a critical obstacle to tissue regeneration. Under stress conditions such as inflammation, leakage of mtDNA persistently activates this pathway, producing excessive inflammatory factors like IFN-I, which directly impair the regenerative capacity of stem cells. For example, in periodontitis, the inflammatory microenvironment causes mtDNA from periodontal ligament stem cells (PDLSCs) to leak through the mitochondrial permeability transition pore (mPTP), abnormally activating cGAS-STING and thereby damaging their osteogenic differentiation ability, hindering alveolar bone regeneration [175]. In intervertebral disc degeneration, hypoxia and other stresses activate the cGAS-STING pathway, inducing ferroptosis in stem cells and accelerating tissue degradation [176]. Furthermore, in senescent nucleus pulposus mesenchymal stem cells (NPMSCs), reduced expression of the epigenetic regulator EZH2 relieves the transcriptional repression of STING, leading to excessive pathway activation, driving the SASP, creating an inflammation microenvironment that inhibits regeneration, and accelerating disc degeneration [177]. This “senescence-inflammation” axis is also a key driver of failed tissue regeneration in neurodegenerative diseases [178]. Interference with key regenerative signaling pathways is another means by which the cGAS/STING pathway impedes regeneration. Research further reveals that cGAS-STING activation can directly suppress the Hippo-YAP pathway—a core regenerative signal regulating cell proliferation and organ size [179]. In endothelial cells, whether due to inflammatory injury or metabolic stress (such as palmitic acid induction), cGAS-STING activation inhibits YAP, impairing endothelial cell proliferation and angiogenic capacity, thereby hindering tissue repair [179, 180].

Therefore, in this context, inhibiting the cGAS-STING pathway becomes a strategy to promote regeneration. Examples include developing biomaterial carriers for the local sustained release of STING inhibitors (e.g., C-176) to protect transplanted stem cells, or employing smart hydrogel systems to locally reconstruct cGAMP metabolic homeostasis and relieve the suppression of PDLSCs [175, 176].

In contrast to the destructive effects of chronic activation described above, acute and controlled activation of cGAS-STING has been shown to be necessary for initiating regenerative programs in specific contexts. This role is most directly evidenced in peripheral nervous system injury repair. After peripheral nerve axon injury, neurons themselves secrete interferon-gamma (IFN γ), actively upregulate cGAS expression, and produce cGAMP [181]. This endogenous, timely triggered pathway activation has been proven to effectively coordinate the innate immune responses of neurons and non-neuronal cells (such as macrophages), creating a favorable microenvironment for axon regeneration and serving as a key driver of spontaneous axon regeneration. This indicates that the pathway can be carefully “orchestrated” to serve repair goals.

In summary, the cGAS-STING pathway presents a clear, unified paradigm in regenerative medicine: chronic, uncontrolled activation (typically originating from persistent organelle damage and mtDNA leakage) is the “foe” of regeneration, whereas acute, self-limiting activation (as a precise response to acute injury) can be the “friend.” The dual role of the cGAS-STING pathway in regenerative medicine perfectly echoes its “friend-or-foe” dialectical relationship in cancer immunity. It reaffirms that this pathway is a core regulatory node for maintaining cellular homeostasis, not a simple “on/off” switch. Regenerative medicine and cancer therapy, from two seemingly opposing directions, jointly point to the ultimate challenge and opportunity in future therapeutics: performing spatiotemporally specific and precise fine-tuning of the cGAS-STING pathway according to the specific “context” of the disease (injury type, stage, cellular environment), thereby transforming destructive inflammatory signals into constructive forces that promote repair or immune attack.

## Conclusion

The cGAS-STING pathway plays a complex and multifaceted role in cells. One paradox is, what is the significance of the DNA sensor cGAS being present in the nucleus? Although our review has provided some reasons for the existence of nuclear cGAS, its purpose in the nucleus has not yet been fully elucidated. It may have other key biological functions beyond its role as a cytoplasmic DNA sensor and may play other roles in biological evolution. Another paradox is whether cGAS/STING is friend or foe; in our view, it depends entirely on context. Crucially, the balance between the “friend” and “foe” phenotypes is dictated by the interplay of signal origin, strength, duration, and microenvironment. Acute, high-intensity activation of the pathway—often induced by therapeutic agents like radiotherapy or targeted STING agonists—predominantly in antigen-presenting cells (APCs) such as dendritic cells, robustly engages the IRF3/type I interferon axis. This initiates a productive immune cycle: DC maturation, cross-priming of tumor-specific CD8⁺ T cells, and enhanced NK cell cytotoxicity, collectively establishing an immunostimulatory microenvironment conducive to tumor control. In stark contrast, chronic, low-grade activation—frequently stemming from intrinsic tumor features like persistent CIN and subsequent cytosolic DNA leakage—often originates within the tumor cells themselves. This sustained signal leads to cellular adaptation and signal rewiring. Key adaptations include the desensitization of the beneficial IFN-I response and the preferential engagement of alternative downstream branches, such as the non-canonical NF-κB pathway. These shifts promote processes that define the “foe” phenotype: upregulation of PD-L1, secretion of immunosuppressive factors (e.g., IL-35 from B cells), induction of Tregs, inhibition of NK-cell activity, and ultimately, the facilitation of metastasis and an immunosuppressive tumor microenvironment.

Therefore, the cGAS-STING pathway does not harbor an intrinsic pro- or antitumor nature. Instead, it functions as a highly context-dependent cellular signaling hub. Its ultimate role in cancer is decided by the tumor’s specific biological context, which shapes the signal’s origin and the network’s output.

In therapy, some studies have shown that slow activation of the cGAS-STING pathway in certain tumors actually promotes tumor deterioration, indicating the potential application of cGAS inhibitors in advanced tumors [182]. In summary, tumor type, stage of development, state of CIN, and the microenvironment may all be potential factors affecting the response of tumor cells to cGAS/STING agonists or antagonists. Therefore, a deeper understanding of the determinants driving the heterogeneity of cGAS/STING signaling in tumors is critical, and personalized treatment strategies tailored to individual patients should be developed on the basis of these insights. Future research needs to delve into the exact mechanisms and downstream effects of the cGAS-STING pathway in tumor development from multiple perspectives, as well as its interactions with other signaling pathways. Moreover, developing more refined and personalized diagnostic and therapeutic strategies targeting the cGAS-STING pathway will also be a key focus of future research.
